# Unveiling the Effect of Scanning Speed on the Corrosion and Tribological Performance of Electron Beam Melted (EBM) Ti-6Al-4V-ELI Alloy

**DOI:** 10.3390/ma18235367

**Published:** 2025-11-28

**Authors:** Eurico Felix Pieretti, Davide Piaggio, Renato Altobelli Antunes, Mara Cristina Lopes de Oliveira, Luís Carlos Elias da Silva, Camila Ramos Silva, Tania Mateus Yoshimura, Wagner de Rossi, Martha Simões Ribeiro, Maurício David Martins das Neves

**Affiliations:** 1 Nuclear and Energy Research Institute, São Paulo 05508-000, Brazil; lcesilva@ipen.br (L.C.E.d.S.); camilaramos.silva@gmail.com (C.R.S.); wderossi@ipen.br (W.d.R.);; 2 School of Engineering, University of Warwick, Coventry CV4 7AL, UK; 3 Center for Engineering, Modeling and Applied Social Sciences (CECS), Federal University of ABC (UFABC), Santo André 09210-580, Brazil

**Keywords:** Ti-6Al-4V-ELI, nanotribology, corrosion, electron beam melting, biomaterials, scanning speed

## Abstract

The influence of electron beam melting (EBM) scan speed on the corrosion, nano-biotribological, and cellular adhesion properties of Ti-6Al-4V-ELI (extra low interstitials) was systematically investigated. Specimens were fabricated using five different scanning speeds, and tribological performance was assessed via reciprocating dry wear tests, while corrosion behaviour was evaluated through monitoring the open circuit potential and anodic potentiodynamic polarization tests in Ringer’s solution. Human fibroblasts from the FN1 cell line were used to assess cell adhesion. Specimens produced using scanning speeds of 4530 mm·s^−1^ and 4983 mm·s^−1^ exhibited increased passive current densities, indicating reduced corrosion protection, although all surfaces maintained the passive film characteristic. Tribological behaviour was strongly dependent on scan speed, with wear rate and penetration depth increasing at higher speeds; notably, an intermediate scan speed produced a surface with minimal wear and penetration depth despite a wide wear track, suggesting enhanced resistance to tribological degradation. Fibroblast cultures demonstrated robust adhesion and spindle-shaped morphology across all samples, with the disk produced using a scanning speed of 4983 mm·s^−1^ showing the highest surface coverage, highlighting the role of EBM process parameters in modulating surface properties relevant to cell–biomaterial interactions. These findings underscore the critical influence of scan speed on the multifunctional performance of Ti-6Al-4V-ELI for biomedical applications.

## 1. Introduction

Metallic biomaterials are widely used in medical implants and devices due to their excellent mechanical properties, biocompatibility, and corrosion resistance [1,2,3,4,5]. These materials, such as stainless steel, titanium alloys, and cobalt-chromium alloys, are commonly used for applications like orthopaedic implants, dental implants, and cardiovascular stents. Despite their advantageous properties, they face challenges such as corrosion, wear, and mechanical failures during prolonged use in the human body [6,7,8,9]. Understanding these challenges is critical to ensuring the long-term performance and safety of these biomaterials.

Currently, 3D printing techniques, such as electron beam melting (EBM) and selective laser melting (SLM), are widely used for manufacturing implant devices. Both EBM and SLM processes can modify the microstructure and surface finishing of the material, which affects its tribological properties. EBM uses an electron beam to melt and solidify the metallic powder, creating a dense microstructure with fine grain boundaries. SLM, a laser-based process, uses a high-power laser to melt the metal powder layer by layer, resulting in a more complex surface finishing with different roughness. These differences necessitate detailed tribological and electrochemical testing to understand the material’s performance under in vivo conditions.

Corrosion is a major concern for metallic biomaterials, as it affects their durability, mechanical integrity, and biocompatibility [10,11,12,13]. In the body’s physiological environment, metallic biomaterials are exposed to highly corrosive body fluids that can lead to the degradation of the material.

Alloys like ASTM F139 stainless steel, Ti-6Al-4V, and Co-Cr-Mo form a protective oxide layer that shields the underlying metal from corrosion [4,12]. Any disruption of this oxide layer, due to mechanical damage or chemical exposure, can lead to corrosion. Additionally, the pH, chloride concentration, and oxygen levels in body fluids affect the material’s corrosion rate. The presence of chloride ions is particularly damaging as it can lead to pitting corrosion or crevice corrosion [14,15].

Wear is the process by which material is gradually removed from the surface of metallic biomaterials due to frictional forces during mechanical contact. In the case of implants such as artificial joints or dental implants, wear is an inevitable process, and wear particles can influence the surrounding tissues. Wear can cause surface roughening, which can accelerate corrosion and affect the overall biocompatibility of the material, generating debris that can lead to inflammation, fibrosis, and osteolysis (bone resorption) in the surrounding tissues [16,17].

Failures in metallic biomaterials can occur due to a combination of corrosion, wear, and mechanical overload. These failures can significantly affect the performance of implants and lead to complications such as implant loosening, infection, or even device failure. Repeated loading can induce fatigue cracks in metallic biomaterials, particularly in areas where stress concentrations exist, such as at the interface of the implant and bone. The presence of tensile stresses and corrosive ions, especially chloride, can lead to stress corrosion cracking, particularly in stainless steels and titanium alloys [18,19,20,21,22].

When produced by additive manufacturing (AM), the properties of Ti-6Al-4V ELI alloys can differ from conventionally manufactured materials due to the nature of the AM process, which involves layer-by-layer deposition of material. The microstructure, surface roughness, and porosity of AM-produced alloys can influence their tribological behaviour (friction, wear, and lubrication).

Sharma et al. [23] studied the dry sliding wear behaviour of Ti–6Al–4V alloy components produced by EBM, comparing heat-treated samples with as-built ones. Water-quenched samples exhibited the least wear loss, while furnace-cooled samples showed the highest wear loss and the lowest microhardness. Most samples experienced material loss due to abrasion.

Gayathri et al. [24] assessed the biocorrosion resistance of SLM Ti-6Al-4V alloy, finding a corrosion rate of 9 × 10^−4^ mm/year in simulated body fluid. The analysis showed the formation of calcium hydroxyapatite, similar to human bone material, indicating good biocompatibility for implant use.

The tribocorrosion behaviour of Ti6Al4V samples produced via selective laser melting was studied by Toptan et al. [25] in comparison with their hot-pressed (HP) and commercial counterparts in 9 g/L NaCl solution at body temperature. The results indicated that tribocorrosion did not show any statistically significant difference in total volume loss or volume loss due to mechanical wear and wear-accelerated corrosion among the processing routes.

There is limited information in the literature regarding the corrosion and biotribological performance of Ti-6Al-4V alloy produced by EBM, as well as the influence of process parameters, such as scanning speed, on this behaviour, focusing on topics at the interface of the biomedical sciences and materials engineering.

This research aimed to investigate the effect of the scanning speed on the tribological performance of EBM-manufactured Ti-6Al-4V-ELI for biomedical applications, with an emphasis on friction, wear, and electrochemical behaviour. Additionally, the influence of scanning speed on cellular adhesion to the Ti-6Al-4V-ELI disk surfaces was evaluated.

## 2. Materials and Methods

### 2.1. Material and Sample Preparation

The Ti-6Al-4V-ELI (extra low interstitials) powder, purchased from AP&C Inc., Boisbriand, QC, Canada, was used to obtain five samples in disk format with 15 mm in diameter, and 3 mm in high, by EBM technique, at 5 different scanning speed parameters: (1) 4077, (2) 4300, (3) 4530, (4) 4757, (5) 4983 [mm·s^−1^]. This numeric sample code will be used throughout the whole text. The purpose of changing the scanning speed parameter is to produce implantable devices faster, without energy loss, with lower operator costs, and less time using the equipment. The powder chemical composition, particle size distribution, flow rate, and apparent and tap densities are shown in Table 1. The equipment used was an Arcam Q10 plus EBM 3D printer machine (Arcam, Mölndal, Sweden), with print space dimensions: 200 mm × 200 mm × 180 mm, electron beam with a spot size of 100 μm generated at a voltage of 60 kV, and a beam power of 6.2 W. The layer thickness was set to 50 μm, and the build plate was preheated to 554 °C. Hardness measurements for all samples yielded values of 353 ± 25 HV. Surface preparation of all specimens involved sequential mechanical grinding up to 2400 grit using silicon carbide (SiC) waterproof abrasive papers, followed by polishing with 3 µm Al_2_O_3_ suspension. The samples were subsequently cleaned in an ultrasonic bath with deionized water for 5 min and air-dried under ambient conditions. The polished specimens were chemically etched using Kroll reagent by applying a cotton swab saturated with a solution composed of 5 mL HF, 30 mL HNO_3_, and 65 mL H_2_O. The etchant was manually rubbed onto the polished surface for approximately 20–30 s to reveal the microstructural features.

Several statistical parameters can be derived from a particle size distribution (D). Among the most important parameters are certainly the percentiles. These indicate in each case the size below which a certain quantity of the sample lies. The particle size distribution of powders is typically expressed by these three values, i.e., D10, D50, and D90.

### 2.2. Tribological and Electrochemical Tests

The electrochemical experiments were conducted in a potentiostat/galvanostat (Iviumn n-Stat, Eindhoven, The Netherlands). A conventional three-electrode cell arrangement was used: the metallic disk as the working electrode, a platinum wire as the counter-electrode, and an Ag/AgCl (KCl, 3 M) electrode as the reference. The electrolyte was Ringer’s physiological solution, pH 7.4, at 25 °C, containing 8.6 g·L^−1^ NaCl, 0.3 g·L^−1^ KCl, and 0.33 g·L^−1^ CaCl_2_. The open circuit potential vs. time was monitored for 15 min (900 s), and then the linear potentiodynamic polarization (LP) measurement was performed at a scan rate of 1.0 mV·s^−1^. All the electrochemical tests were repeated six times (n = 6) in order to confirm their reproducibility.

The tribological trials were assessed by reciprocating dry wear tests conducted in a nanotribometer (Anton Paar—model NTR^2^, Graz, Austria), performed in the air, at 25 °C, with counter-body of chrome steel 52-100 rotating ball shape, 825 HV in hardness, 2 mm in diameter, for 10 min, with normal force of 100 mN, sliding distance equivalent to 2.4 m, and scan speed of 4.0 cm·s^−1^. The surfaces were all electrolytically polished in a solution of perchloric acid and ethanol before testing. All tracks were made in the same region of the sample that was subjected to the polarization tests. To confirm its reproducibility, six wear tracks were made on each surface.

### 2.3. Surface Morphology

A LEXT OLS4100 (Olympus™, Tokyo, Japan) confocal laser scanning microscope (CLSM) was used to acquire topographical images of the EBM Ti-6Al-4V-ELI samples. Line-profile roughness measurements were obtained using a 50× objective and processed using the analysis modules provided in the LEXT software (https://www.olympus-ims.com, accessed on 10 October 2023).

The surface characterization was conducted using a scanning electron microscope (SEM-EDX), model TM3000 (Hitachi, Tokyo, Japan), and a scanning electron microscope (SEM-EDS) JSM-IT700HR (Jeol, Tokyo, Japan).

### 2.4. Cell Culture and Adhesion on Ti-6Al-4V-ELI Disks

Normal human fibroblast cells (FN1) were isolated from a patient, characterized, and cultivated by Prof. Dr Durvanei A. Maria at the Butantan Institute in São Paulo, Brazil. These cells represent a typical fibroblast morphology and a duplication time of approximately 24 h. The cell line is registered at the Clinical Hospital of the Medical School, University of São Paulo (HCFMUSP nº 921/06).

Human fibroblasts from the FN1 cell line were maintained under cell culture conditions until reaching confluence. An aliquot of 50 µL (2.0 × 10^4^ cells/µL) was placed at the centre of the Ti-6Al-4V-ELI disks and kept under cell culture conditions for approximately 3 h to allow for cell adhesion. After this period, 3 mL of culture medium was added to the wells of a 6-well plate in which the disks were placed, and the setup was maintained for 48 h in an incubator at 37 °C. Following the incubation period, the disks were gently washed with buffer solution and immersed in a 4% paraformaldehyde solution for 30 min to fix the cells.

Standardized images from different regions of each disk were captured using a polarized light microscope (Leica DMLP, Buffalo Grove, IL, USA). To analyze the percentage of surface area covered by cells on each disk, ImageJ software (https://imagej.nih.gov, accessed on 20 May 2025) was used with the “Analyze Particles” plugin.

Data normality was assessed using the Shapiro–Wilk test, and homogeneity of variances was evaluated with Levene’s test in OriginPro 2018. Comparisons among disks were performed using one-way ANOVA followed by Tukey’s post hoc test. Differences were considered statistically significant at *p* < 0.05 [26,27].

## 3. Results and Discussion

### 3.1. Topographical Characterization

**Figure 1:**
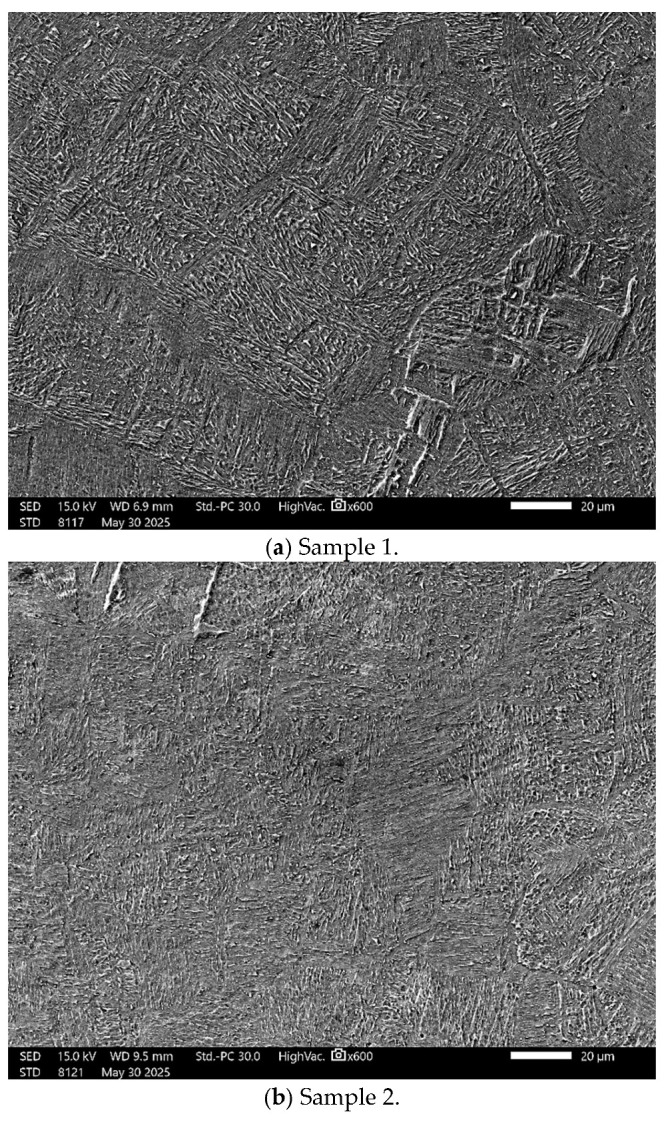

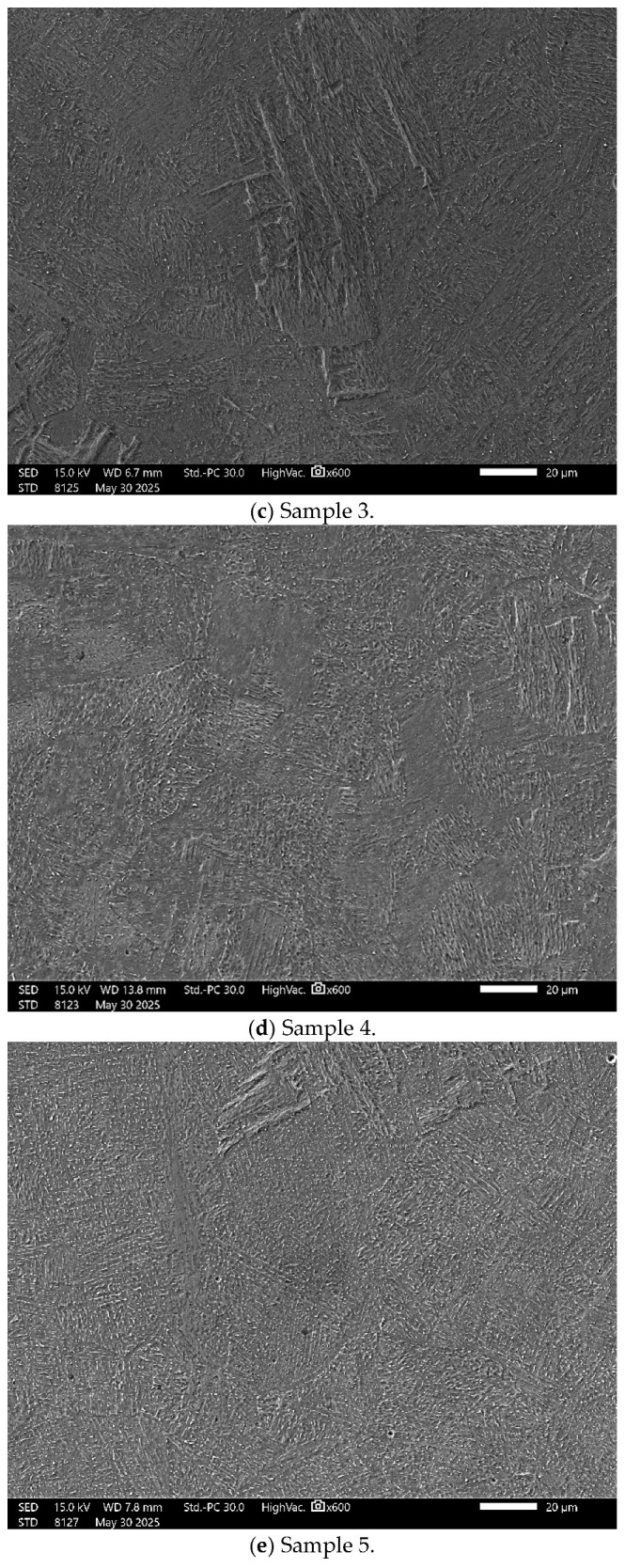

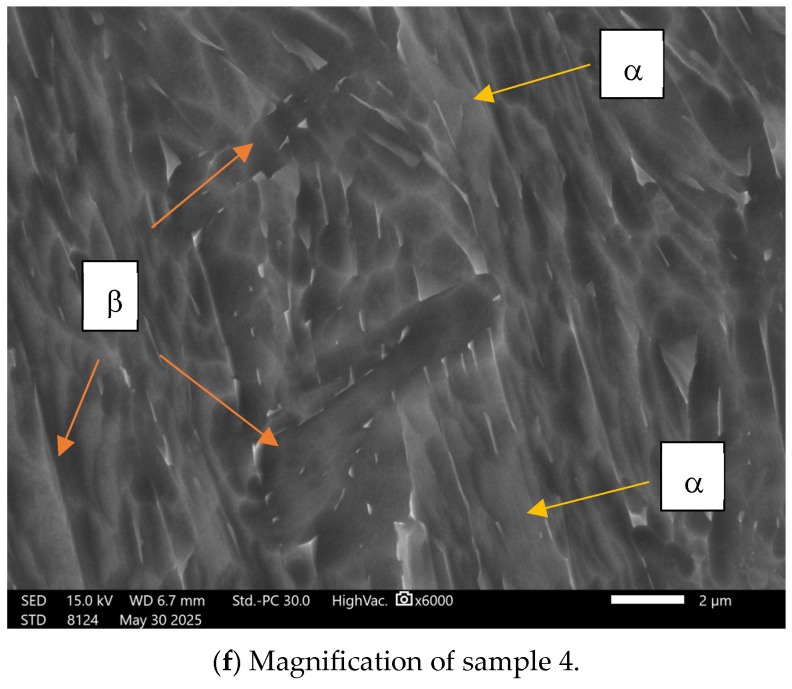
Scanning electron microscopy (SEM) images of Ti-6Al-4V ELI specimens fabricated via electron beam melting (EBM) under different beam scanning speeds, following chemical etching to expose the underlying microstructural features. (**a**) 4077 mm·s^−1^, (**b**) 4300 mm·s^−1^, (**c**) 4530 mm·s^−1^, (**d**) 4757 mm·s^−1^, (**e**) 4983 mm·s^−1^, and (**f**) higher-magnification view of the microstructure shown in (**d**).

Figure 1a–f presents scanning electron microscopy (SEM) micrographs of Ti-6Al-4V-ELI specimens fabricated under varying scanning speed parameters, presenting both polished and chemically etched surfaces to reveal the microstructural features. Figure 1f is a higher-magnification view of sample 4, showing alpha and beta phases and their distribution on the consolidated samples.

The microstructural heterogeneities observed in Figure 1a–f, including variations in grain size and crystallographic orientation, are attributed to the steep thermal gradients and rapid cooling rates characteristic of the EBM process. Figure 1a–e illustrate the topographical variations observed in the specimens fabricated under different electron-beam scanning speeds. The resulting microstructural diversity originates from non-uniform heat dissipation associated with directional solidification during layer-by-layer processing [28]. The microstructure of Ti-6Al-4V ELI fabricated via EBM is characterized by elongated, columnar prior-β grains oriented along the build direction, containing a fine α + β Widmanstätten morphology. The α phase primarily forms as thin lamellae within a retained β matrix, reflecting the solidification and repeated thermal cycling inherent to the layer-by-layer fabrication process, which also contributes to pronounced microstructural anisotropy [28]. At the higher-magnification view shown in Figure 1f, the microstructure reveals a light-grey α-phase matrix containing elongated, parallel α lamellae arranged in a well-developed Widmanstätten morphology. Narrow dark-grey interlamellar films correspond to retained β. Columnar features aligned with the build direction are also evident, indicative of epitaxial β-grain growth during solidification. In EBM processing, the scanning speed governs the energy input to the powder bed and consequently exerts a strong influence on the resulting surface roughness.

**Figure 2:**
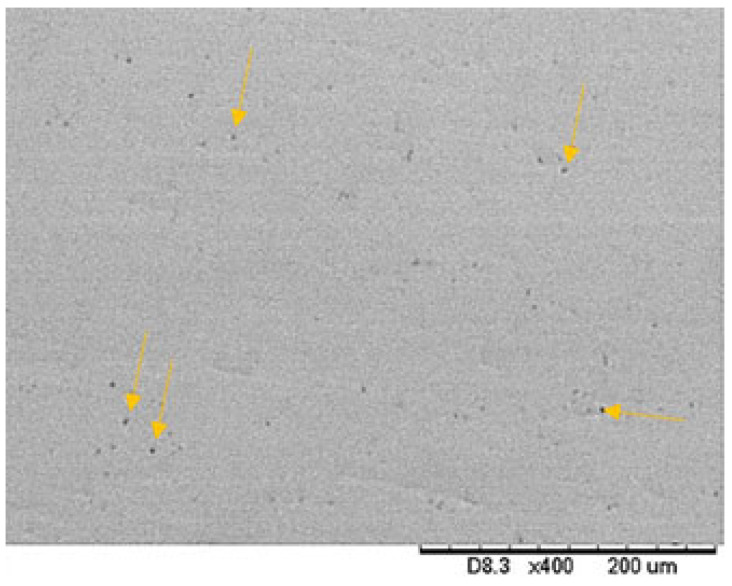
SEM micrograph of the EBM Ti-6Al-V4-ELI specimen after SiC paper grit, without etching. The topography is relatively smooth with some pores (indicated by arrows) due to non-melted powders.

Roughness parameters of each surface type are specified in Table 2. The scanning electron micrographic image of a ground specimen tested in this study can be observed in Figure 2.

As shown in Figure 2, smooth titanium alloy surfaces presented low undulations, which are typical of the grinding process with SiC papers. All five evaluated surfaces showed similar morphology. The surface finishing has been standardized for all samples. Although roughness is nearly similar for all analyzed conditions, it remains a considerable factor that leads to variations in nanotribological behaviour.

The roughness parameters presented in Table 2 are: Ra (arithmetic mean of the absolute departures of the roughness profile from the mean line), and Rq (root mean square parameter corresponding to Ra). All the obtained values are in accordance with those reported in the literature for titanium alloys used for biomedical applications [29]. The lowest Ra value was obtained for samples 1 and 2, whereas the highest ones were for samples 4 and 5, respectively. Since the surface finish was the same for all samples, no significant differences in terms of roughness were expected.

The surface roughness of Ti-6Al-4V-ELI, as measured by Ra and Rq, plays a pivotal role in its tribological and biological performance. These results indicate that samples with higher roughness (e.g., Sample 4) exhibited increased wear rates and penetration depths, aligning with findings by Aswar et al. [30], who reported that surface roughness significantly influences the wear behaviour of Ti-6Al-4V alloys. Their study demonstrated that increased roughness leads to higher friction and wear rates, which aligns with the findings of the present work.

Lower scanning speeds increase the energy input. Higher scanning speeds reduce the energy input per unit length; the beam may not fully melt or bond the uppermost layer, causing loose or semi-sintered particles to remain attached [30].

### 3.2. Electrochemical Tests

Electrochemical measurements are important to provide an estimate of the corrosion susceptibility of biomaterials produced with different additive manufacturing parameters. The passive film formed on the Ti-6Al-4V alloy plays a dominant role in the improvement of corrosion resistance. When the alloy is in contact with a simulated body fluid, the formation of a less defective and more homogeneous passive film is triggered [31,32].

**Figure 3:**
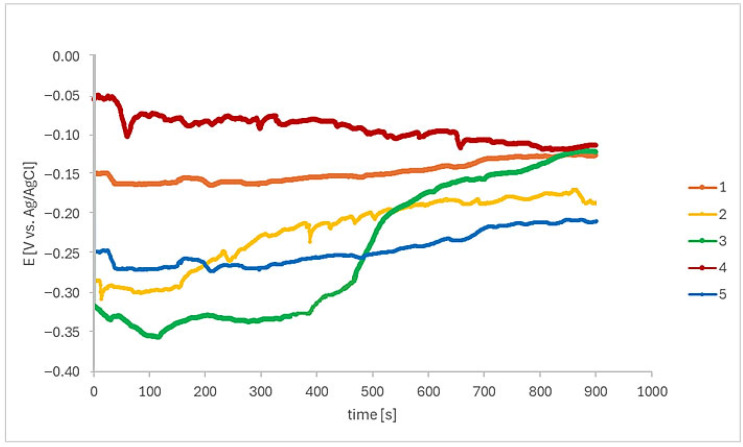
Variation in the OCP with the immersion time in Ringer’s solution for all EBM-manufactured Ti-6Al-4V ELI samples. Total time: 900 s (n = 6).

Initially, the open circuit potential (OCP) was monitored in Ringer’s solution during 900 s, as shown in Figure 3.

Following the immersion period of EBM Ti-6Al–4V-ELI in Ringer’s solution, an E_ocp_ shift towards a positive direction, suggesting a protective oxide layer begins to grow on samples 1 and 2. Then, E_ocp_ remains at a relatively stable potential of −0.14 V/_Ag/AgCl_ and −0.18 V/_Ag/AgCl_, respectively, and then stabilizes. Sample 3 showed E_ocp_ around −0.32 V/_Ag/AgCl_ at the beginning of the monitoring period and then shifted to nobler potentials. Sample 4 showed an E_ocp_ decreasing tendency throughout the test, and sample 5 potentials remained reasonably stable during the test, at E_ocp_ around −0.25 V/_Ag/AgCl_.

The OCP fluctuations are explained by the instantaneous competition between the protective film formation and dissolution [33]. The passive film in these regions is still uneven in the electrolyte, and the appearance of micro-pits leads to a decrease in the potential, which may be attributed to the micron defects formed during the EBM process. Conversely, the broken passive films are re-passivated quickly, indicating an increase in the potential.

**Figure 4:**
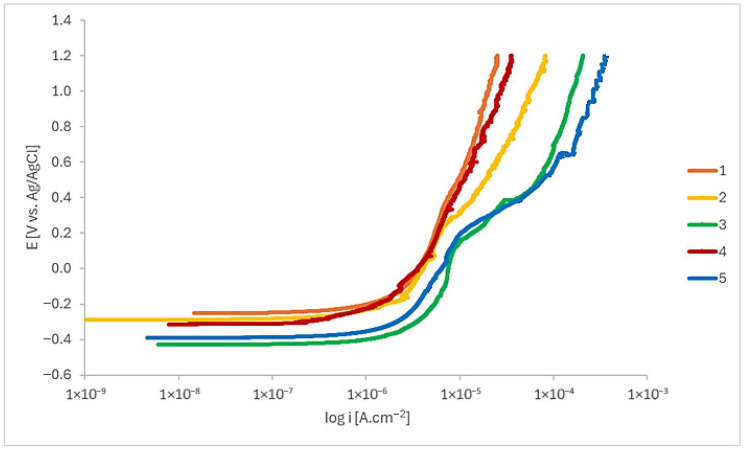
Linear potentiodynamic polarization curves of the EBM-manufactured Ti-6Al-4V ELI samples after 15 min of immersion in Ringer’s solution (n = 6).

Figure 4 shows the anodic linear potentiodynamic polarization curves of the EBM-manufactured Ti-6Al-4V ELI samples after 15 min of immersion in Ringer’s solution. Low corrosion current density (i_corr_) indicates low corrosion rate or high corrosion resistance [34,35].

Samples 1, 2, and 4 exhibited comparable polarization behaviour. In contrast, samples 3 and 5 demonstrated potential deviations at approximately 0.1 V/_Ag/AgCl_ and 0.5 V/_Ag/AgCl_. All curves are characterized by a well-defined passive region. These findings are consistent with the reported passive film for Ti-6Al-4V alloys, which is primarily composed of TiO_2_ and Al_2_O_3_ [36,37,38]. Considering that the surface finishing conditions were similar, this suggests that the scanning speed used in the production of the samples influenced their electrochemical behaviour. The parameters used for samples 3 and 5 resulted in higher passive current densities, suggesting reduced protection.

Wear and corrosion processes are additional effects arising from the interaction between metallic biomaterials and the body tissues [39]. Metallic biomaterials’ properties were evaluated by Okazaki [40] concerning the effect of friction on the electrochemical anodic polarization. Metallic particles released from the corrosion process may move passively, through tissue and/or circulatory system, or can be actively transported [41,42], compromising the implant’s biocompatibility.

### 3.3. Tribological Behaviour

**Figure 5:**
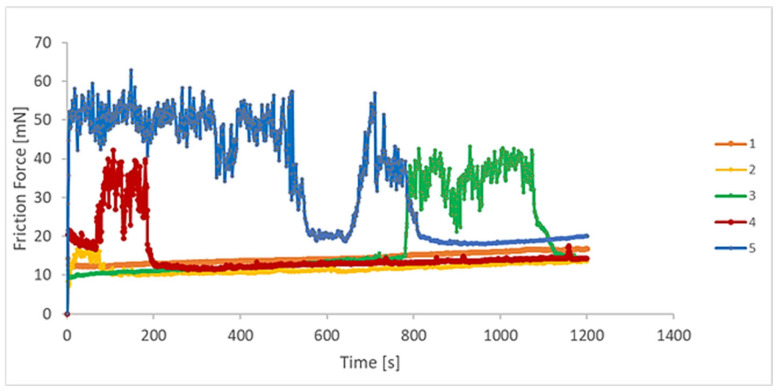
Friction force variation as a function of the sliding time for the EBM-manufactured Ti-6Al-4V ELI samples, without lubrication (n = 6).

**Figure 6:**
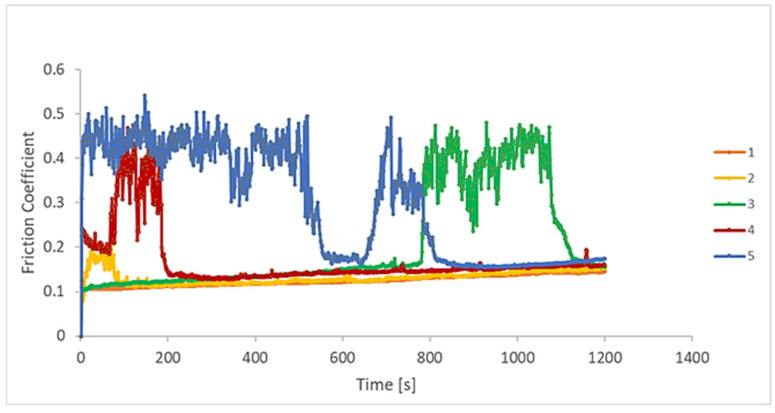
COF as a function of time for the EBM-manufactured Ti-6Al-4V ELI samples, without lubrication (n = 6).

**Figure 7:**
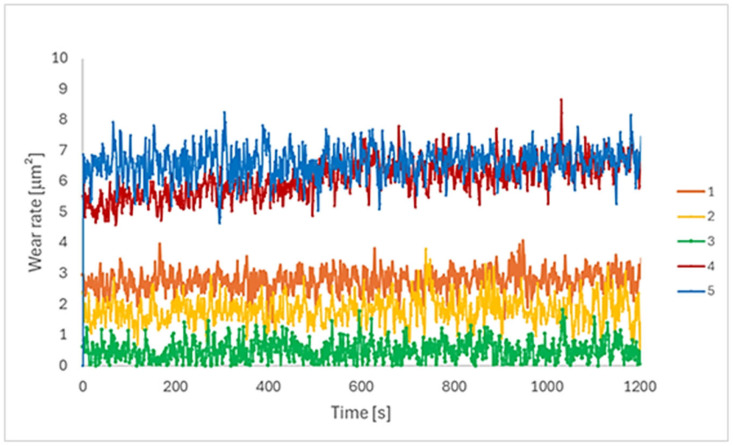
Wear rate [mm^2^] of the EBM-manufactured Ti-6Al-4V ELI samples, without lubrication (n = 6).

**Figure 8:**
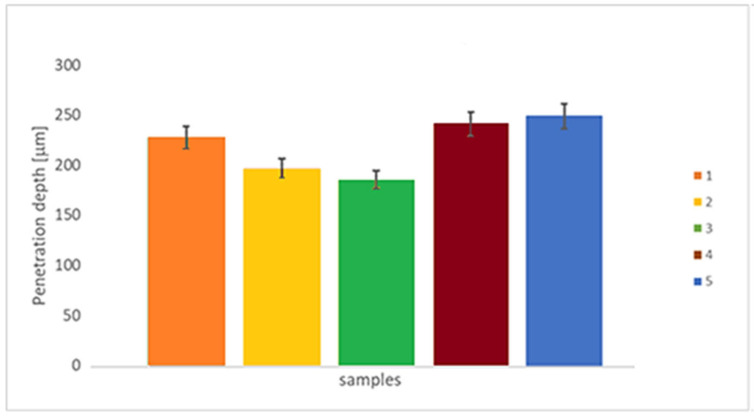
Penetration depth [mm] of the EBM-manufactured Ti-6Al-4V ELI samples, subjected to reciprocating wear tests, without lubrication (n = 6).

Tests in micro and nanotribometers are used to investigate small regions and thin layers of different surfaces. The evolution of the coefficient of friction (COF), friction force, wear rate, and penetration depth were evaluated by nanotribometer reciprocating dry wear tests, as presented in Figure 5, Figure 6, Figure 7 and Figure 8. A relationship between the wear rate and friction coefficient was observed, i.e., the highest value of wear rate was related to the highest value of the coefficient of friction. For the first period of evaluation, samples 5 and 4 presented the highest COF values, respectively. In the case of sample 5, the COF decreases at around 580 s, reaching a stabilization period, then increasing and decreasing again, presenting a stable behaviour up to the end of the test. Samples 1 and 2 remained stable throughout the whole experiment, as the surfaces became less rough. Sample 3 presented stable values from the start until about 800 s. Next, they increased and returned to the initial values at about 1100 s.

The COF variation as a function of the test time was studied by Huang et al. [43]. They verified this effect on the tribological properties of Ti-6Al-4V alloys with and without laser cladding coatings, for a running time of 3500 s in different rotation frequencies. At the end of the tests, they verified that the COF for the coatings was always inferior to the substrate. Typical oscillations of the COF during the steady-state regime of the metal-on-metal sliding contacts are explained based on the third-body effect [44].

The evolution of the wear rate during sliding of the EBM-manufactured Ti-6Al-4V ELI specimens is shown in Figure 6. The wear rate values for all samples showed a practically stable evolution during the test. It can be observed that for all the different processing scanning speeds, the wear rate attains the steady-state regime after a very short running-in period. Similar results were found in tribocorrosion tests for the same Ti alloy, varying the EBM scanning speed [45].

Samples 4 and 5 presented the highest values throughout the test, followed by samples 1 and 2. Sample 3 showed the lowest wear rate values. These results are interesting in terms of the scan speed parameters used in the EBM process. As mentioned previously, the intermediate scan speed value was employed on sample 3, while the other samples were defined as a percentage of (−5% and 10% above and below the scanning speed of sample 3). Hence, the intermediate scanning speed value (sample 3) presented the inferior wear rate. Lower speeds (samples 1 and 2) showed higher wear rates, and higher parameters (samples 4 and 5) showed the highest values.

Figure 8 presents the penetration depth assessed on the tribological test. This value is intended as a spot with the largest distance from the surface reached during the test. The lowest values were found for sample 3, followed by samples 2 and 1, and finally, samples 4 and 5. In terms of the scan speed parameter used on EBM manufacturing, it can be inferred that the medium scan speed value produces samples with a lower penetration depth, while the other conditions produce samples with higher penetration depth values.

Regarding the nanotribological mechanisms, the worn surfaces of all the EBM Ti-6Al-4V ELI samples exhibited plastic deformation as a result of abrasive wear by the incidence of grooves aligned along the sliding path. Similar results were found by Bartolomeu et al. [46] on the wear behaviour of Ti-6Al-4V biomedical alloys processed by selective laser melting, hot pressing, and conventional casting, which showed an influence of the processing route on the microstructural constituents and consequent differences in hardness and wear performance.

**Figure 9:**
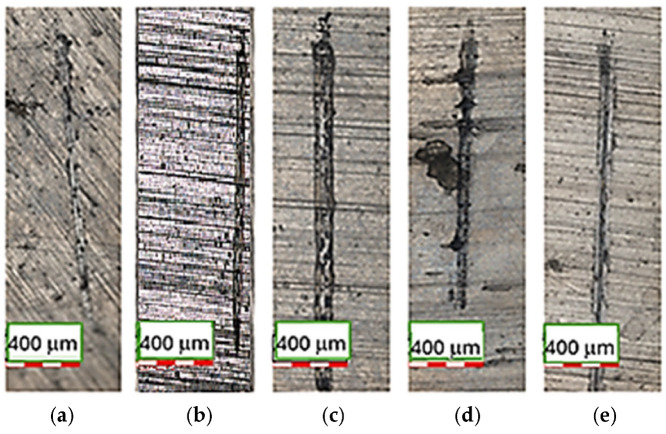
CLSM micrographs of the EBM-manufactured Ti-6Al-4V ELI samples, after electrochemical tests, showing the wear tracks generated after reciprocating sliding tests. The scale bar represents 400 µm: (**a**) 1; (**b**) 2; (**c**) 3; (**d**) 4; (**e**) 5.

The wear tracks of EBM Ti-6Al-4V ELI disks formed after the reciprocating sliding tests against chrome steel 52–100 balls, without lubrication, were analyzed by means of CLSM, as shown in Figure 9a–e.

The wear path generated on the surfaces after electrochemical tests under the same nanotribometer configuration presented distinct dimensions, i.e., different width and length, which is evident in these images, due to the tracks being generated in a different direction from the EBM direction.

According to Figure 7 and Figure 8, sample 3 exhibited the lowest wear rate and penetration depth, despite having a wide wear track. The intermediate electron beam scan speed produced a surface that is less prone to tribological degradation. Since this nanotribological test is non-conformational, the minimal penetration into the surface and the widened path indicate that the rotating solid sphere is more readily consumed than the surface being tested.

The scanning speed employed during EBM processing critically governs the thermal history of Ti-6Al-4V ELI, thereby modulating its microstructural, surface, and functional properties. At lower scanning speeds, the prolonged interaction time between the electron beam and powder bed promotes slower cooling rates, leading to coarser α lamellae and increased residual stresses, which can locally enhance galvanic activity and reduce wear resistance. Conversely, excessively high scanning speeds induce steep thermal gradients and rapid solidification, generating finer α + β Widmanstätten structures but also increasing porosity and microstructural heterogeneity, which can induce heterogeneity in the passive oxide layer, leading to elevated corrosion current densities and concomitant alterations in nanoscale tribological performance [9,38].

The use of a nanotribometer to evaluate the tribological properties of EBM Ti-6Al-4V-ELI is crucial for understanding the performance of these materials in biomedical applications. These devices provide precise measurements of wear, friction, normal force, coefficient of friction, and penetration depth, which are essential for predicting the durability and functionality of implants. By leveraging such technologies and detailed scientific studies, manufacturers can optimize Ti-6Al-4V ELI for use in implants, ensuring improved patient outcomes and implant longevity.

### 3.4. Cell Adhesion Tests

**Figure 10:**
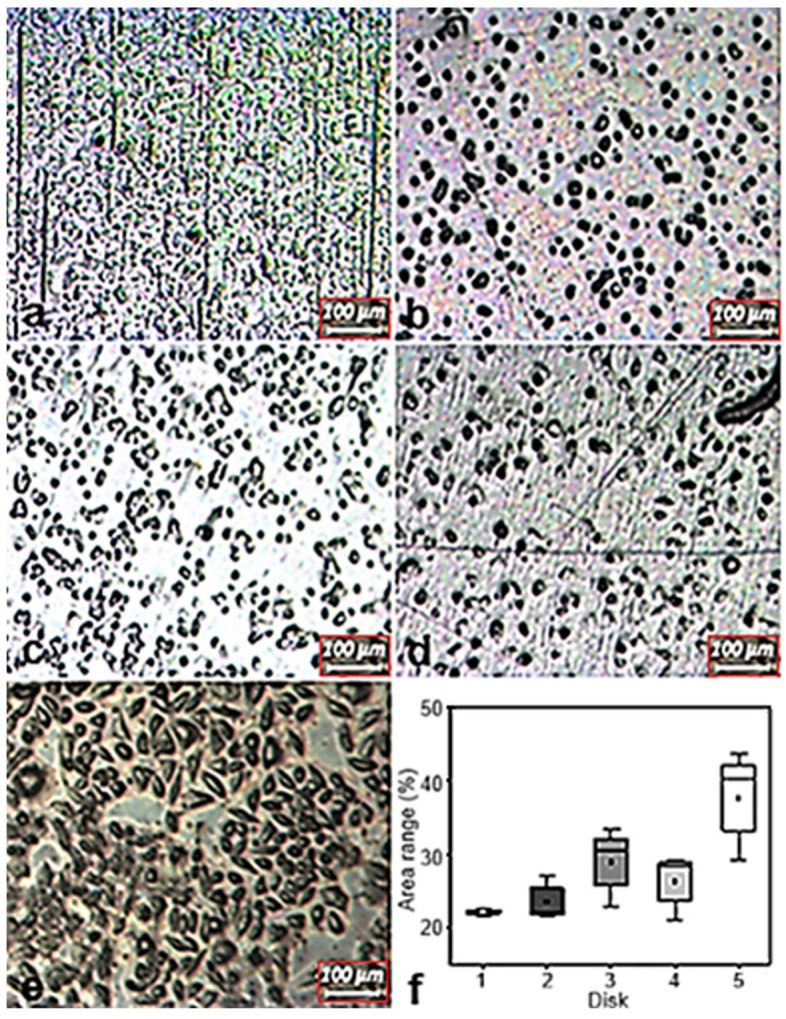
Representative photomicrographs showing cell adhesion on Ti-6Al-4V ELI disks: 1 (**a**), 2 (**b**), 3 (**c**), 4 (**d**), and 5 (**e**). (**f**) Percentage of surface area covered by adhered cells.

Fibroblasts cultured on Ti-6Al-4V-ELI disks exhibited favourable adhesion, as evidenced by their spread morphology and uniform distribution across the substrate (Figure 10a–e). Light microscopy showed predominantly spindle-shaped cells, characteristic of healthy fibroblasts, demonstrating robust cell–surface interactions and confirming the material’s biocompatibility [47]. Furthermore, disk 5 exhibited the highest percentage of covered area, with statistically significant differences compared to disks 1 and 2, as shown in Figure 10f, indicating that variations in EBM scanning speed during manufacturing can influence surface properties relevant to cell adhesion.

Surfaces exhibiting higher corrosion activity may release ions (Ti, Al, V), thereby creating less favourable conditions for cell attachment or modifying local cellular responses. Accordingly, improved electrochemical stability is generally associated with enhanced cytocompatibility [45].

Wear testing provides insight into the surface’s resistance to mechanical interaction; modest micro-roughness can promote cell adhesion by increasing available surface area and enabling mechanical interlocking. In contrast, the generation of metallic or oxide wear particles may elicit inflammatory reactions that indirectly diminish cell adhesion or viability. Additionally, wear tracks that disrupt the passive film can expose fresh metal, altering the surface chemistry. Thus, low wear rates combined with minimal debris formation support the maintenance of a stable and biocompatible surface [48].

The microstructure and surface topography of EBM-fabricated Ti-6Al-4V ELI provide favourable sites for cell adhesion and proliferation. In addition, the hierarchical microstructural features and surface characteristics significantly influence the material’s electrochemical and nanotribological behaviour. Localized galvanic interactions arising from microstructural heterogeneity modulate corrosion performance, while wear resistance and other nanotribological properties are strongly governed by the interplay between phase distribution and surface texture.

## 4. Conclusions

The performance of Ti-6Al-4V-ELI fabricated via electron beam melting (EBM) is strongly influenced by surface topography, which modulates both tribological and electrochemical behaviour. Surfaces with slightly higher roughness may increase contact area and local stress during dry wear, resulting in higher penetration depths and wear rates, whereas smoother surfaces reduce frictional resistance and wear, as reflected by the lower penetration depths observed for samples fabricated at an intermediate scanning speed. These findings suggest that small variations can meaningfully affect surface interactions and biomechanical durability at the nanoscale.

Electrochemical behaviour was also affected by EBM scanning speed, with samples produced using scanning speeds of 4530 mm·s^−1^ and 4983 mm·s^−1^ exhibiting higher passive current densities, suggesting reduced protective efficacy.

Fibroblast cultures on Ti-6Al-4V-ELI demonstrated robust adhesion and typical spindle-shaped morphology, confirming the biocompatibility of the EBM-fabricated material. Notably, the disk produced with a scanning speed of 4983 mm·s^−1^ exhibited significantly higher cell coverage compared to disks produced with 4077 mm·s^−1^ and 4300 mm·s^−1^, indicating that variations in EBM scanning speed can modulate surface features that influence cellular attachment. These results highlight that careful optimization of EBM process parameters provides a viable strategy to enhance surface characteristics and promote favourable cell–material interactions for biomedical applications.

## Figures and Tables

**Table 1:** Ti-6Al-4V-ELI (ASTM F30001) powder characteristics.

Chemical Composition [wt%]		
Ti	Al	V
Bal.	6.34 ± 0.05	3.98 ± 0.02
Particle size distribution (ASTM B822)		
D10 [mm]	D50 [mm]	D90 [mm]
51.0 ± 0.03	69.0 ± 0.04	96.2 ± 0.03
Flow rate [50 g·s^−1^]	Apparent density [g·cm^−3^]	Tap density [g·cm^−3^]
25.0 ± 0.03	2.6 ± 0.01	2.8 ± 0.03

**Table 2:** Roughness parameters of EBM Ti-6Al-4V-ELI specimens measured by CLSM.

Surface	Ra [μm]	Rq [μm]
1	0.303 ± 0.172	0.425 ± 0.412
2	0.320 ± 0.175	0.440 ± 0.413
3	0.326 ± 0.115	0.518 ± 0.308
4	0.618 ± 0.181	0.711 ± 0.199
5	0.501 ± 0.173	0.587 ± 0.323

## Data Availability

The data presented in this study are available on request from the corresponding author due to ongoing research.

## References

[1] Pieretti E.F., Pessine E.J., Correa O.V., Rossi W., Neves M.D.M. (2015). Effect of Laser Parameters on the Corrosion Resistance of the ASTM F139 Stainless Steel. Int. J. Electrochem. Sci..

[2] Zhang L.C., Liu Y., Li S., Hao Y. (2017). Additive Manufacturing of Titanium Alloys by Electron Beam Melting: A Review. Adv. Eng. Mater..

[3] Pieretti E.F., Costa I. (2013). Surface characterization of ASTM F139 stainless steel marked by laser and mechanical techniques. Electrochim. Acta.

[4] Kurtz M.A., Wessinger A.C., Mace A., Moreno-Reyes A., Gilbert J.L. (2023). Additively manufactured Ti-29Nb-21Zr shows improved oxide polarization resistance versus Ti-6Al-4V in inflammatory simulating solution. J. Biomed. Mater. Res. A.

[5] Assis S.L., Costa I. (2007). Electrochemical evaluation of Ti-13Nb-13Zr, Ti-6Al-4V and Ti-6Al-7Nb alloys for biomedical application by long-term immersion tests. Mater. Corros..

[6] Assis S.L., Wolynec S., Costa I. (2006). Corrosion characterization of titanium alloys by electrochemical techniques. Electrochim. Acta.

[7] Hedberg Y.S., Žnidaršič M., Herting G., Milošev I., Wallinder I.O. (2019). Mechanistic insight on the combined effect of albumin and hydrogen peroxide on surface oxide composition and extent of metal release from Ti6Al4V. J. Biomed. Mater. Res. B Appl. Biomater.

[8] Bordbar-Khiabani A., Gasik M. (2023). Electrochemical and biological characterization of Ti-Nb-Zr-Si alloy for orthopedic applications. Sci. Rep..

[9] Bocchetta P., Chen L.-Y., Tardelli J.D.C., Reis A.C., Almeraya-Calderón F., Leo P. (2021). Passive Layers and Corrosion Resistance of Biomedical Ti-6Al-4V and β-Ti Alloys. Coatings.

[10] Fangaia S.I.G., Messias A., Valente A.J.M., Guerra F.A.D.R.A., Nicolau P.M.G. (2024). Evaluation of the Tribocorrosion Behavior of Ti-6Al-4V Biomedical Alloy in Simulated Oral Environments. Processes.

[11] Lavrys S., Pohrelyuk I., Veselivska H., Skrebtsov A., Kononenko J., Marchenko Y. (2022). Corrosion behavior of near-alpha titanium alloy fabricated by additive manufacturing. Mater. Corr..

[12] Jáquez-Muñoz J.M., Gaona-Tiburcio C., Baltazar-Zamora M.A., Méndez-Ramírez C.T., Estupinán-López F., Bautista-Margulis R.G., Cuevas-Rodríguez J., Flores-De los Ríos J.P., Almeraya-Calderón F. (2023). Corrosion of Titanium Alloys Anodized Using Electrochemical Techniques. Metals.

[13] Fernandes Santos P., Niinomi M., Liu H., Cho K., Nakai M., Trenggono A., Champagne S., Hermawan H., Narushima T. (2016). Improvement of microstructure, mechanical and corrosion properties of biomedical Ti-Mn alloys by Mo addition. Mater. Des..

[14] Williams D.F. (1976). Corrosion of Implant Materials. Mater. Sci..

[15] Gibbons D.F. (1975). Biomedical Materials. Biophys. Bioeng..

[16] Bertolini R., Ghiotti A., Bruschi S. (2021). Wear Behavior of Ti6Al4V Surfaces Functionalized through Ultrasonic Vibration Turning. J. Mater. Eng. Perform..

[17] Hammood A.S., Thair L., Altawaly H.D., Parvin N. (2019). Tribocorrosion Behaviour of Ti–6Al–4V Alloy in Biomedical Implants: Effects of Applied Load and Surface Roughness on Material Degradation. J. Bio-Tribo-Corr..

[18] Seo D.I., Lee J.B. (2023). Localized corrosion and repassivation behaviors of additively manufactured titanium alloys in simulated biomedical solutions. npj Mater. Degrad..

[19] Boraei N.F.E., Ibrahim M.A.M., Rehim S.S.A.E., Elshamy I.H. (2024). Electrochemical corrosion behavior of β-Ti alloy in a physiological saline solution and the impact of H_2_O_2_ and albumin. J. Solid State Electrochem..

[20] Ednie L., Antonysamy A.A., Parimi L., Mani M., Thomas M., Lancaster R.J. (2024). Understanding the fatigue behaviour of Ti–6Al–4V manufactured via various additive processes. J. Mater. Res. Technol..

[21] Mohazzab B.F., Jaleh B., Fattah-Alhosseini A., Mahmoudi F., Momeni A. (2020). Laser surface treatment of pure titanium: Microstructural analysis, wear properties, and corrosion behavior of titanium carbide coatings in Hank’s physiological solution. Surf. Inter..

[22] Pou P., Riveiro A., Val J., Comesaña R., Penide J., Arias-González F., Sotoa R., Lusquiños F., Pou J. (2017). Laser surface texturing of titanium for bioengineering applications. Procedia Manuf..

[23] Sharma D., Kamran M., Paraye N.K., Anant R. (2021). Insights into the wear behaviour of electron beam melted Ti–6Al–4V alloy in the as-built and the heat-treated conditions. J. Manufact. Process.

[24] Gayathri Y.K.B., Kumar R.L., Ramalingam V.V., Priyadharshini G.S., Kumar K.S., Prabhu T.R. (2022). Additive Manufacturing of Ti-6Al-4V alloy for Biomedical Applications. J. Bio-Tribo-Corr..

[25] Toptan F., Alves A.C., Carvalho O., Bartolomeu F., Pinto A.M.P., Silva F., Miranda G. (2019). Corrosion and tribocorrosion behaviour of Ti6Al4V produced by selective laser melting and hot pressing in comparison with the commercial alloy. J. Mater. Process. Tech..

[26] Han Y., Xing X., Zhou L., Huang S., Lin Z., Hong G., Chen J. (2024). GL13K-modified titanium regulates osteogenic differentiation via the NF-κB pathway. Int. Immunopharmacol..

[27] Shapiro S.S., Wilk M.B. (1965). An analysis of variance test for normality (complete samples). Biometrika.

[28] Somlo K., Poulios K., Funch C.V., Niordson C.F. (2021). Anisotropic tensile behaviour of additively manufactured Ti-6Al-4V simulated with crystal plasticity. Mech. Mater..

[29] Ponader S., Vairaktaris E., Heinl P., Wilmowsky C.V., Rottmair A., Körner C., Singer R.F., Holst S., Schlegel K.A., Neukam F.W. (2007). Effects of topographical surface modifications of electron beam melted Ti-6Al-4V titanium on human fetal osteoblasts. J. Biomed. Mater. Res. Part A.

[30] Aswar S.J., Chakule R.R., Baviskar D., Khandare N.H., Kharche Y.A., Deshmukh D.M., Dakhole M.Y., Vishwanatha S., Aden A.A. (2025). Enhancing surface finish and increasing fatigue resistance of Ti6Al4V produced through electron beam melting via chemical machining. J. Mater. Sci. Mater. Eng..

[31] Li J., Li S.J., Hao Y.L., Yang R. (2014). Electrochemical characterization of nanostructured Ti–24Nb–4Zr–8Sn alloy in 3.5% NaCl solution. Int. J. Hydrogen Energy.

[32] Tang J., Luo H.Y., Zhang H.B. (2017). Enhancing the surface integrity and corrosion resistance of Ti-6Al-4V titanium alloy through cryogenic burnishing. Int. J. Adv. Manuf. Technol..

[33] Hodgson A.W.E., Mueller Y., Forster D., Virtanen S. (2002). Electrochemical characterisation of Passive Films on Ti Alloys Under Simulated Biological Conditions. Electrochim. Acta.

[34] Hou X., Ren Q., Yang Y., Cao X., Hu J., Zhang C., Deng H., Yu D., Li K., Lan W. (2021). Effect of temperature on the electrochemical pitting corrosion behavior of 316L stainless steel in chloride-containing MDEA solution. J. Nat. Gas Sci. Eng..

[35] Jafarzadegan M., Ahmadian F., Salarvand V., Kashkooli S. (2020). Investigation of microstructure and corrosion resistance of AISI 304 stainless steel joint with ER308 and ERNiCr-3 filler metals by GTAW. Metall. Res. Technol..

[36] Reséndiz L.R., Cano T.S., Naeem M., Rehman A.U., Salamci E., Mendoza V.T., Duran E.D., Díaz L.B., Salamci M.U. (2024). Mechanical and Electrochemical Properties Comparison of Additively Manufactured Ti-6Al-4V Alloys by Electron Beam Melting and Selective Laser Melting. J. Mater. Eng. Perform..

[37] Jaquez-Muñoz J., Gaona-Tiburcio C., Liramartínez A., Zambrano-Robledo P., Maldonado-Bandala E., Samaniego-Gamez O., Nieves-Mendoza D., Olguín-Coca J., Estupiñán-López F., Almeraya-Calderón F. (2021). Susceptibility to Pitting Corrosion of Ti-CP2, Ti-6Al-2Sn-4Zr-2Mo, and Ti-6Al-4V Alloys for Aeronautical Applications. Metals.

[38] Tardelli J.D.C., Bolfarini C., Reis A.C. (2020). Comparative Analysis of Corrosion Resistance between Beta Titanium and Ti-6Al-4V Alloys: A Systematic Review. J. Trace Elem. Med. Biol..

[39] Liu Y., Tan G., Tang J., Zhang L., Shen G., Gu Z., Jie X. (2023). Enhanced corrosion and wear resistance of Zn–Ni/Cu–Al_2_O_3_ composite coating prepared by cold spray. J. Solid State Electrochem..

[40] Okasaki Y. (2002). Effect of friction on anodic polarization properties of metallic biomaterials. Biomaterials.

[41] Black J. (1984). Systemic effects of biomaterials. Biomaterials.

[42] Anderson J.M. (2001). Biological response to materials. Annu. Rev. Mater. Res..

[43] Huang C., Zhang Y., Vilar R., Shen J. (2012). Dry sliding wear behavior of laser clad TiVCrAlSi high entropy alloy coatings on Ti-6Al-4V substrate. Mater. Des..

[44] Doni Z., Alves A.C., Toptan F., Gomes J.R., Ramalho A., Buciumeanu M., Palaghian L., Silva F.S. (2013). Dry sliding and tribocorrosion behaviour of hot-pressed CoCrMo biomedical alloy as compared with the cast CoCrMo and Ti6Al4V alloys. J. Mater. Des..

[45] Pieretti E.F., Silva L.C.E., Ribeiro M.S., de Rossi W., Antunes R.A., Oliveira M.C.L., Piaggio D., Cruz F.P.A., da Paz J.O., das Neves M.D.M. (2025). Triboelectrochemical Performance of Electron Beam–Melted Ti-6Al-4V-ELI for Biomedical Applications. Adv. Mater. Sci. Eng..

[46] Bartolomeu F., Buciumeanu M., Pinto E., Alves N., Silva F.S., Carvalho O., Miranda G. (2017). Wear behavior of Ti6Al4V biomedical alloys processed by selective laser melting, hot pressing, and conventional casting. Trans. Nonferr. Met. Soc. China.

[47] Kulkarni M., Mazare A., Gongadze E., Perutkova Š., Kralj-Iglič V., Milošev I., Schmuki P., Iglič A., Mozetič M. (2015). Titanium nanostructures for biomedical applications. Nanotechnology.

[48] Podlipec R., Punzón-Quijorna E., Pirker L., Kelemen M., Vavpetič P., Kavalar R., Hlawacek G., Štrancar J., Pelicon P., Fokter S.K. (2021). Revealing Inflammatory Indications Induced by Titanium Alloy Wear Debris in Periprosthetic Tissue by Label-Free Correlative High-Resolution Ion, Electron and Optical Microspectroscopy. Materials.

